# Construction of high-density genetic linkage map and identification of flowering-time QTLs in orchardgrass using SSRs and SLAF-seq

**DOI:** 10.1038/srep29345

**Published:** 2016-07-08

**Authors:** Xinxin Zhao, Linkai Huang, Xinquan Zhang, Jianping Wang, Defei Yan, Ji Li, Lu Tang, Xiaolong Li, Tongwei Shi

**Affiliations:** 1 Department of Grassland Science, Sichuan Agricultural University, Chengdu, 611130, China; 2 Agronomy Department, University of Florida, FL, 32610, USA; 3 Biomarker Technologies Corporation, Beijing, 101300, China

## Abstract

Orchardgrass (*Dactylis glomerata* L.) is one of the most economically important perennial, cool-season forage species grown and pastured worldwide. High-density genetic linkage mapping is a valuable and effective method for exploring complex quantitative traits. In this study, we developed 447,177 markers based on SLAF-seq and used them to perform a comparative genomics analysis. Perennial ryegrass sequences were the most similar (5.02%) to orchardgrass sequences. A high-density linkage map of orchardgrass was constructed using 2,467 SLAF markers and 43 SSRs, which were distributed on seven linkage groups spanning 715.77 cM. The average distance between adjacent markers was 0.37 cM. Based on phenotyping in four environments, 11 potentially significant quantitative trait loci (QTLs) for two target traits–heading date (HD) and flowering time (FT)–were identified and positioned on linkage groups LG1, LG3, and LG5. Significant QTLs explained 8.20–27.00% of the total phenotypic variation, with the LOD ranging from 3.85–12.21. Marker167780 and Marker139469 were associated with FT and HD at the same location (Ya’an) over two different years. The utility of SLAF markers for rapid generation of genetic maps and QTL analysis has been demonstrated for heading date and flowering time in a global forage grass.

Orchardgrass (*Dactylis glomerata* L.) is one of the top four most economically important perennial forage grasses and is native to northern Africa, Europe, and temperate Asia^1^,^2^. Tetraploid orchardgrass is the most widespread among the more than 200 cultivars currently available^1^, and it has been naturalized on almost every continent due to its extensive uses for forage, hay, and pasture^3^,^4^. Flowering time (FT) is key to the productivity of many flowering plants^5^. Early-flowering cultivars tend to have higher seed yield, while late-flowering cultivars are desirable in pastures because livestock avoid consuming flowering stems^6^. Late-heading date cultivars are also used when mixing orchardgrass with legumes^7^,^8^. However, because of the large genome and autotetraploid status of orchardgrass, it is difficult for growers and breeders to easily identify the genes controlling FT. Therefore, revealing the placement of FT control genes in linkage groups and developing species-specific markers for marker-assisted selection (MAS) is of significant value in breeding programs.

MAS is in high demand in molecular breeding programs for its independence from the environment and high efficiency for selection of desirable lines. Construction of high-density molecular genetic maps and identification of quantitative trait loci (QTLs) are critical steps for MAS. For example, 23 traits, including developmental, morphological, and phenological traits, were detected based on the linkage maps of two major tetraploid switchgrass cultivars (Alamo-A4 and Kanlow-K5)^9^. High-density genetic maps for two diploid *Miscanthus sinensis* cultivars were produced, and three QTLs for zebra stripe intensity (zbi1, zbi2, and zbi3 on linkage groups 7, 10, and 3) were identified^10^. A genetic map with 434 restriction-site associated DNA (RAD) markers and simple sequence repeat (SSR) markers in perennial ryegrass allowed the concentrations of palmitic, stearic, linoleic, and a-linolenic acids to be identified^11^. The high-density genetic maps of *Zoysia japonica* were compared with those of other species and provided insights into genome evolution in the Chloridoideae^12^.

In the past few years, much progress has been made in the construction of genetic markers and linkage maps in orchardgrass. The first orchardgrass map was constructed using 164 SSRs and 108 sequence-related amplified polymorphism (SRAP) markers for two Chinese diploid cultivars, 01996 and YA02-103, with average distances of 9.6 cM in the male map and 8.9 cM in the female map^13^. More recent orchardgrass linkage maps were constructed for tetraploid orchardgrass based on either SSR or amplified fragment length polymorphism (AFLP) markers with relatively low marker density^14^,^15^. Among these markers, SSRs are the most widely used due to their conservation, synteny, and superior transferability^16^. Recently, 606 polymorphic SSR markers were developed from a Japanese orchardgrass variety, Akimidori II^2^,^17^. Orchardgrass expressed sequence tag (EST) libraries constructed from three cultivars–Latar, Paiute, and Potomac–were used to develop SSR markers pertaining to salt, drought, and cold stress^17^. Though some species-specific orchardgrass markers were developed^17^,^18^, the number of traditional genetic markers is still very limited for high-density map construction.

As next-generation sequencing (NGS) technology has become available, it is now possible to achieve dense marker coverage without a reference genome^19^. For example, NGS-based technologies such as RAD-seq^20^ and specific-locus amplified fragment sequencing (SLAF-seq)^21^ have recently been developed and could provide an ample number of markers for high-density genetic map construction. These methods have been used in many species for map construction, including the use of RAD-seq in perennial ryegrass^11^, and the use of SLAF-seq in soybean^22^, sesame^23^, cucumber^24^, and walnut^25^. Combining NGS and reduced representation libraries (RRL), SLAF-seq is a very time- and cost-effective method. The efficiency of SLAF-seq was tested on data from rice and soybean. Results showed that the marker arrangement and order were consistent between the map and genome in rice, with the genetic map consisting of 12 linkage groups corresponding to the 12 rice chromosomes^26^. A consistent locus on Gm13 was detected by QTL mapping and genome-wide association study (GWAS) mapping approaches in soybean^27^. SLAF-seq was demonstrated to be an ideal tool with high resolution for large-scale genotyping in QTL mapping or gene discovery studies. In this study, we constructed a high-density genetic map by combining SLAF and SSR markers, and we identified QTLs associated with heading date (HD) and FT using these markers. The result is, to our knowledge, the densest genetic map in orchardgrass. In addition, our analysis was based on field evaluations over two years and at two different locations. This map should serve as a platform for future identification and genetic dissection of many other complex and important traits.

## Results

### Analysis of SLAF-seq data

In total, we obtained 291.21 M reads after SLAF library construction and high-throughput sequencing. The average percentage of Q30 bases (bases with a quality score of 30, indicating a 1% chance of an error and thus 99% confidence) was 89.4% for all reads. The average guanine-cytosine (GC) content was 45%. *Arabidopsis thaliana* (Columbia ecotype, genome size = 119.48M) was used as a control to estimate the validity of library construction. We generated 93,742 total *A. thaliana* reads with 89.29% Q30 bases and 46.46% GC content (Supplemental Table 1). A total of 301,898 and 303,836 SLAFs were generated for the orchardgrass male and female parents, respectively. The average number of SLAFs in the progeny was 172,334 (Supplemental Table 2). We detected 447,177 high-quality SLAF markers in total. These SLAF markers could be separated into three groups: 89,038 polymorphic markers (including mapped biallelic markers and unmapped biallelic markers), 8,777 repetitive markers (mutiallelic markers with tag number larger than 4 in parents) and 349,362 non-polymorphic markers (monomorphic markers with only one tag in parents) (Supplemental Table 3). Mutiallelic SLAFs which could not be used for recombination rate calculating were removed from further analysis. Of the 89,038 polymorphic SLAF markers, 52,600 biallelic markers were further classified into eight segregation patterns (Supplemental Fig. 1). The CP (common parent) population was obtained from the F1 hybrid offspring of two fully homozygous parents. Therefore, only the aa × bb segregation pattern, which included 15,891 SLAF markers, could not be used to construct the genetic map. The remaining 36,709 polymorphic SLAFs were therefore used to construct the genetic map.

### Linkage map construction

**Figure 1:**
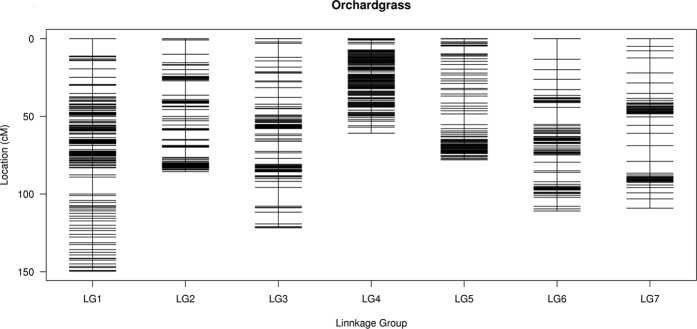
Distribution of SLAF and SSR markers on seven linkage groups. The linkage group number is shown on the x-axis, and genetic distances (cM) are shown on the y-axis.

After a five-step filtering process (see Methods), 2,922 polymorphic SLAFs (Supplementary Table 4) and 114 SSR markers that followed the 1:1 Mendelian segregation pattern were used for constructing the orchardgrass genetic map using HighMap software. Two linkage maps, one for each parent, were constructed. The female map spanned 666.80 cM, with 1,594 markers and an average inter-marker spacing of 0.48 cM (Supplementary Table 5). The length of the male parent map was 658.74 cM, with 1,024 markers and an average inter-marker spacing of 0.89 cM (Supplementary Table 6). A combined outbred linkage map was constructed from the two parent maps using 2,467 SLAFs and 43 SSRs (2,510 total markers) arranged into seven linkage groups (LGs) (Fig. 1, Table 1). The length of the combined map was 715.77 cM, and the average distance between adjacent markers of the map was 0.37 cM. The number of markers in each LG varied from 161 to 688, with an average of 359 markers per LG. The sizes of the individual LGs ranged from 60.85 to 149.66 cM, with average inter-marker distances of 0.19–0.68 cM. LG1 was the longest group, with 688 loci spanning 149.66 cM, while LG4 was the shortest, with 326 loci spanning 60.85 cM. LG4 was also the densest, whereas LG7 contained the fewest markers. The SSR markers were distributed across LG1, LG2, LG3, LG6, and LG7. Several markers were mapped to the same linkage group when comparing our map and our previous tetraploid map^14^ (Supplemental Fig. 2). For example, MarkerOG537N2 and MarkerOG677N2, located in LG1 of that previous tetraploid map, were grouped in A-H1 and L-H1 (A and L are the parents, and H is the combined linkage group). Similarly, MarkerOG365N1 and MarkerOG365N3, located in LG2, were grouped in A-H2, and MarkerOG402N2, located in LG6, was found in L-H6. This result supports that our current high-density linkage map of orchardgrass can be a reliable reference for mapping important traits and for comparing and combining different genetic maps.

### Comparative genome analysis

The 447,177 high-quality SLAF markers generated from orchardgrass were compared with the genome sequences of four other grass species: perennial ryegrass (*Lolium perenne* L.), barley (*Hordeum vulgare* L.), rice (*Oryza sativa* L.), and the model plant species *Brachypodium distachyon*. The total numbers of matching marker containing sequences between orchardgrass and each species were 22,448 (5.02%) to perennial ryegrass; 19,207 (4.30%) to barley; 14,478 (3.24%) to *B. distachyon*; and 5,432 (1.21%) to rice (Table 2). The 2,259 mapped SLAF markers were also assessed in the other four grass species. The mapped markers followed a similar trend to that of all markers, with 124 (5.02%) matching to perennial ryegrass, 73 (2.96%) matching to barley, 63 (2.55%) matching to *B. distachyon*, and 15 (0.61%) matching to rice. These results reflect the fact that orchardgrass is more closely related to perennial ryegrass than to the other three grasses. A Circos plot was used to show the linear relationships between orchardgrass and barley, rice, and *B. distachyon* (Supplemental Fig. 3), illustrating a correspondence between the mapped SLAF markers and their genomic locations. Comparison of the orchardgrass LGs and the barley chromosomes showed that each of the seven LGs was primarily divided into two parts that were distributed across different barley chromosomes. Although there were only 15 mapped markers between orchardgrass and rice, rice chromosomes 1, 2, 4, and 8 with two mapped markers were divided to form LG1, 2, 4, 5, 6 in orchardgrass. For *B. distachyon*, only one marker in LG3 could be identified on chromosome 3 using BLAST. All markers in LGs were also found across different chromosomes in *B. distachyon*. Although 461,845 scaffolds have been assembled in perennial ryegrass, only 590 scaffolds were included in its genetic map^28^. There was no correspondence between the 2,467 mapped orchardgrass SLAF markers and the 590 mapped perennial ryegrass scaffolds.

### Phenotypic variation

**Figure 2:**
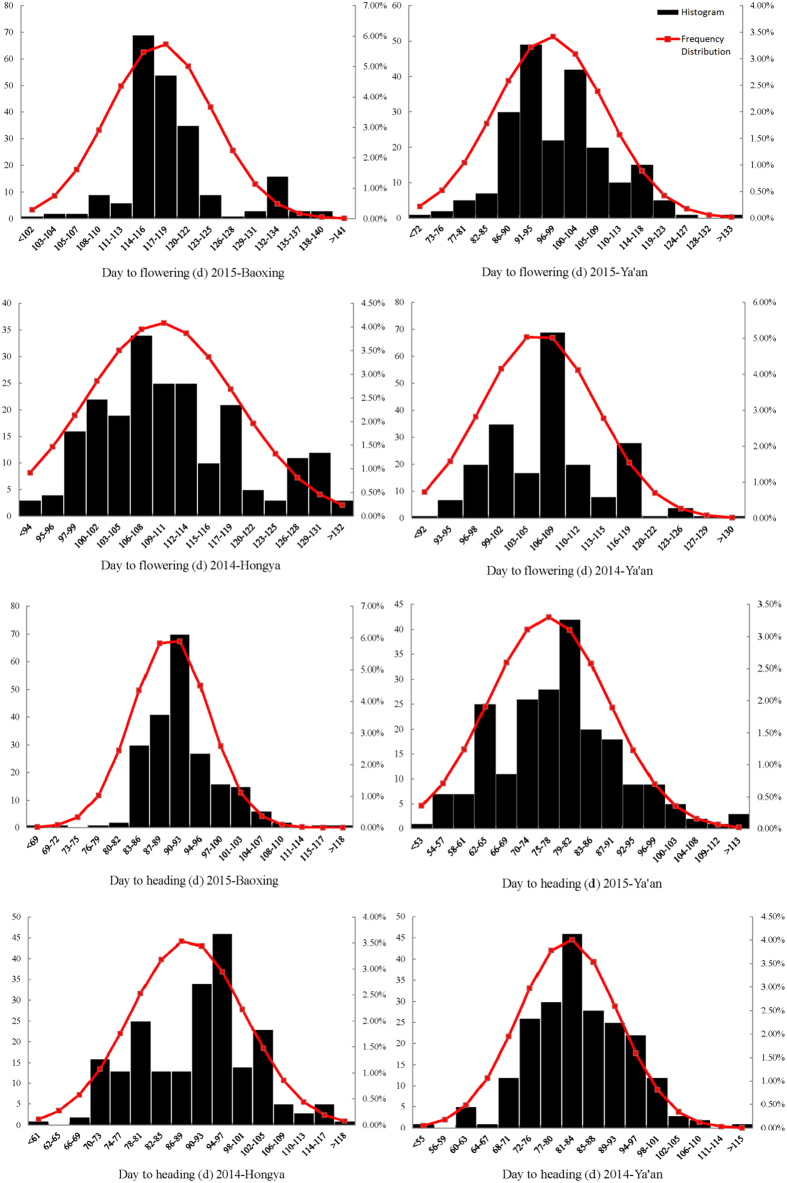
Frequency distributions of orchardgrass heading times and flowering times. The x-axis indicates the days to heading and flowering. The left y-axis indicates the number of individuals, and the right y-axis reflects the frequency distribution.

The FT of the orchardgrass population ranged from 72 to 145 days, with an average of 115 days across the four data sets (two years and two locations). The HD ranged from 53 to 120 days, with an average of 88 days. The shortest average FT was 99 days in data set E2 (2015-Ya’an), and the longest was 118 days in E1 (2015-Baoxing) (Table 3). For HD, the shortest average was 53 days in E2, and the longest was 69 in E1. The results showed that FT and HD exhibited similar trends across the different environments, indicating that the environment had almost the same influence on FT and HD. The coefficients of variation of FT ranged from 5.82% (E1) to 11.75% (E2), and the coefficients for HD ranged from 7.15% (E1) to 12.42% (E3, 2014-Hongya), demonstrating that E1 had the smallest influence on the traits and was more stable for genetic study than the other three environments. The spearman correlation coefficients was analyzed by SAS. The expected high correlation coefficients were calculated between FT and HD (Table 4). The two traits were normally distributed among the population over the two years (Fig. 2).

### QTL analysis

**Figure 3:**
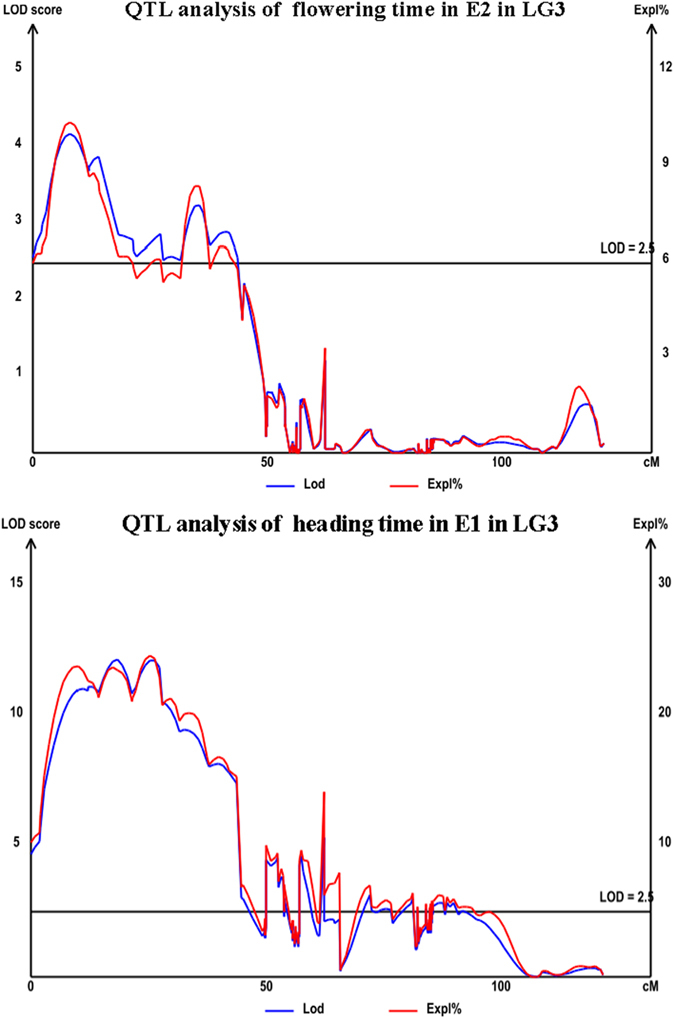
QTL analysis of heading date and flowering time in linkage group 3 (LG3). Logarithm of odds (LOD; blue) and percentage of phenotypic variance explained (Expl%; red) curves for heading time and flowering time of orchardgrass markers (x-axis) in LG3. The gray line indicates the threshold LOD score of 2.5.

An interval mapping model with a LOD (logarithm of odds) score of 2.5 for potential QTLs was used for QTL detection. In total, 11 significant QTLs for HD and FT were found to be distributed on LG1, LG3, and LG5 of the orchardgrass map (Table 5, Fig. 3). For FT, the QTL with the highest LOD score (8.69) was located at 12.01 cM of LG3 near Marker139469 (Table 5). The proportion of phenotypic variation explained by this QTL was 18.30%. For HD, the highest LOD score (12.21) was found at Marker126472, located at 18.31 cM of LG3, and explained 23.60% of the phenotypic variation. Marker167780 and Marker139469 were implicated in both FT and HD at the same experimental location (Ya’an) in both years. In order to explore potential candidate genes, we compared and annotated the significant QTLs for FT and HD. After annotation, Marker250988 was found to belong to the *hd1* gene. One candidate gene, *VRN1 (Lp_74D14_1* and *Lp_7D23_1*), was found by analysis of annotated genes.

## Discussion

SLAF-seq is a recently developed NGS-based genotyping approach, and it has been demonstrated to be highly accurate and cost-effective. SLAF-seq genotyping results have provided important information for genome evolution studies and molecular-assisted breeding. This method has recently been applied successfully to draft genomes^29^ and can be used to study genome evolution^30^ and construct high-density genetic maps in several plant species^31^,^32^,^33^,^34^. Because of its large genome size (4,300 Mb), heterozygosity, and ploidy level, sequencing of the complete orchardgrass genome has been challenging^35^. The low cost and high accurancy of SLAF-seq makes it an approachable method for marker enrichment and molecular plant breeding. Genetic map construction and identification of species-specific markers for MAS are becoming the most feasible methods for development of molecular breeding programs for crop species without a reference genome. Here, we devolped high-throughput markers for orchardgrass. In total, 447,177 SLAF markers were developed, and 2,922 filtered polymorphic markers were eventually identified for genetic linkage map construction. BLAST analysis was first performed between the orchardgrass SLAF markers and the genome sequences of four related grass species, confirming that orchardgrass is more closely related to perennial ryegrass than to the other three grass species. As *Lolium* and *Dactylis* are both in the Pooideae, perennial ryegrass could be regarded as a model plant for further genome analysis in orchardgrass. Orchardgrass was more closely related to *B. distachyon,* which has five chromosomes and belongs to the Festucoideae, than to rice, which has twelve chromosomes and belongs to the Oryzoideae, concurring that the Pooideae are more closely related to the Festucoideae than to the Oryzoideae. *B. distachyon* and barley are different species in same subfamily Festucoideae, and we found that orchardgrass was more closely related to barley than to *B. distachyon.* It has been proposed that the common ancestor of all grass species most likely had seven chromosomes^36^. Following whole-genome duplication (WGD) or polyploidization, chromosome numbers usually decline, and some genetic information may be lost. As a valuable and integral grass species, orchardgrass could be used to study this process in depth in the future.

Genetic maps are also important for genome sequence assembly, QTL mapping, and comparative genomic analyses among grasses. The *Z. japonica* genetic map was also compared with that of other species to provide insights into genome evolution and chromosome number evolution in the Chloridoideae^12^. In our study, comparisons between mapped SLAF markers in orchardgrass and four other genome sequences showed similar trends to the BLAST results from all high-quality SLAF markers. A WGD/polyploidization event occurred before the divergence of the major cereals^37^, reflecting the distant relationship between orchardgrass and rice. Only one marker in LG3 was identified on chromosome 3 of *B. distachyon* with BLAST, whereas Bd21 lacked a member of the CO gene family (*CO3*) and a vernalization pathway gene (*VRN2*)^37^, showing that the genes corresponding to HD and FT QTLs in orchardgrass may not be present in *B. distachyon.* No correspondence was found between the perennial ryegrass and orchardgrass genetic maps, reflecting the fact that the perennial ryegrass genetic map has a low density of markers. *Dactylis* and *Lolium* are core forage and turf grasses. The study and utilization of the Pooideae are becoming more prevalent in grass research. However, this is the first time that the SLAF-seq technique was successfully applied to the development of markers in a forage grass with high specificity and stability.

A high-density genetic linkage map, containing the largest number of molecular markers among previously reported genetic maps^23^,^24^,^25^, was constructed with the SLAF-seq technique. However, because of the limited size of the mapping population and density of markers, many QTLs for important economic traits cannot be accurately detected. In this study, we identified 2,510 markers, including 2,467 SLAF markers and 43 SSR markers, and grouped them into seven linkage groups for construction of an integrated map. This genetic map, with an average inter-marker distance of 0.37 cM, represents, to our knowledge, the most extensive orchardgrass genetic map to date. This map also has a much higher marker density than most genetic maps constructed with SLAF markers^23^,^34^,^38^,^39^,^40^,^41^,^42^. HighMap software and collinearity with the genome sequence^32^ were used to evalute the quality of previous genetic maps. The marker order and map distances of our map were validated with HighMap^21^. Since the genome of orchardgrass has not been sequenced, the collinearity could not be analyzed. We therefore used SSR markers selected from the first tetraploid orchardgrass genetic map to evaluate the quality of our map. SSR MarkerOG365N1 and MarkerOG365N3, located in LG2, were grouped in A-H2, and MarkerOG402N2, located in LG6, was found in L-H6. These results validate the reliability of our map. However, our genetic map can be further saturated to cover the whole genome with recently enriched orchardgrass-specific markers developed through different methods^2^,^17^,^18^.

Flowering time is an important trait of forage grass cultivars and is strongly affected by energy density and dry matter digestibility^43^. Heading date mainly determines adaptability to regional and environmental conditions and has been a major target in breeding programs^44^. Here, we measured FT and HD in four environments (two locations over two years) and analyzed the phenotypic variation and frequency distribution (Fig. 2). Previous studies showed that FT and HD are complex traits controlled by a network of regulatory genes involved in development and signal transduction^43^. Specifically, vernalization^45^,^46^ and photoperiodicity^47^,^48^ have been reported to affect the genetic and physiological mechamisms of grass FT and HD. One crucial and challenging factor in the genetic analysis of complex traits is the degree of phenotypic variation^49^. This study demonstrated a wide range of phenotypic variation in the FT and HD of orchardgrass in a segregating population in four environments. The shortest average FT and HD were 99 days and 53 days, respectively, in E2, and the longest were 118 days and 69, respectively, in E1 (Table 3). This indicates that the two traits respond similarly to different environments. The range of FT and HD variation showed that E1 had the smallest influence on the traits, as there was a narrower range of variation than in the other three environments. The values of skewness were all nearly 0 for FT and HD at E1–E4, while the values of kurtosis were all around 3. The spearman correlation coefficients was analyzed by SAS which showed that these results indicated accurate phenotyping for QTL analysis (Table 4).

The mapping of QTLs in grasses is challenging, especially for autotetraploid and self-incompatible grasses with high heterozygosity and large genome sizes, such as orchardgrass^35^. Therefore, it is difficult to generate a permanent population and to develop sufficient numbers of molecular markers. The first HD QTL analysis in orchardgrass was reported using the F1 population of a ‘*asch*621’ and ‘*him*271’ cross. Xie *et al*.^15^ conducted a QTL study for HD using SSR and AFLP markers; the sequence region harboring the two flanking SSR markers exhibited homology to physically mapped rice genes and explained 12–24% of the phenotypic variation. With advances in sequencing technology and its application in marker development, significant progress has been made in the analysis of FT and HD QTLs in key crops such as rice^50^, wheat^51^, barley^52^, and maize^53^. In the present study, 11 QTLs associated with HD and FT were found to be distributed across three LGs (LG1, LG3, and LG5). The results showed that the FT QTLs that were detected explained 8.20–27.00% of the phenotypic variance, while the HD QTLs explained 10.10–23.60% (Table 5). In previous research, many major and minor QTLs related to HD were detected in barley at different locations^52^. Similarly, in rice, several different QTLs associated with HD were located across different chromosomes, and Hd1 was identified as a candidate gene by means of a map-based cloning strategy^50^. Five QTLs for vernalization response, measured as days to heading, were identified and mapped to four linkage groups in perennial ryegrass^54^, implicating the gene *VRN1*. These results showed that both HD and FT QTLs could be detected and identified by high-densty map constrution and QTL analysis. In our study, we detected several QTLs associated with HD and FT. After annotation, an HD-related QTL was annotated as belonging to the *hd1* gene, and an FT-related QTL was annotated as belonging to the *VRN1 (Lp_74D14_1* and *Lp_7D23_1*) genes. The fact that these genes are associated with FT and HD is unsurprising, since vernalization has been reported to be associated with flowering and development in previous HD QTL analyses. Markers surrounding these QTLs could be listed as candidate genes for further screening and verification and applied to association analysis for fine mapping. One of the most significant results of this study was the mapping of an HD and FT QTL on LG3 that was consistently found across two different years. Future studies involving the genotyping of this region on LG3 with a handful of existing markers (Marker167780 and Marker139469) would be useful. Marker167780 and Marker139469, detected in all four environments, were not annotated with any related genes. These consistent QTLs with stable and significant effects on the phenotypes of FT and HD may be valuable resources for candidate gene exploration in the future.

## Materials and Methods

### Plant materials and DNA isolation

An F1 population of 213 individuals derived from the cross between two Chinese orchardgrass cultivars–‘Kaimo’ (early flowering time) and ‘01436’ (late flowering time)–was used for map construction. Plants were grown in a research field of the Grassland Science Department, Sichuan Agricultural University in Ya’an, Sichuan, China. Each individual plant in the F1 population was clonally divided in the Baoxing Research Field in the summer of 2013. Two replicates of each of the 213 genotypes in the mapping population and the two parental lines were planted in October of 2013. The first few young of each genotype were collected for DNA isolation. Samples were lyophilized and stored at −20 °C until use. Total genomic DNA was isolated using the Plant Genomics DNA Kit (TIANGEN Biotech) according to the manufacturer’s instructions. The concentration and quality of genomic DNA were tested by agarose gel electrophoresis and ND-1000 Spectrophotometer (NanoDrop).

### SLAF and SSR data analysis and genotyping

Genomic DNA samples of the two parents and 213 F1 progeny individuals were subjected to SLAF-seq with some modifications^21^. Briefly, SLAFs were selected for paired-end sequencing on an Illumina HiSeq 2500 sequencing platform, performed by the Beijing Biomarker Technologies Corporation. The sequencing data obtained in our study has been deposited in the NCBI-short read archive (SRA) database under BioProject accession number (PRJNA321844). All paired-end reads (200 bp per read) generated from SLAF-seq raw reads were clustered according to sequence similarity. Identical reads were merged to avoid repeat computing requirements, and sequences with over 90% identity were grouped into one SLAF locus, as described^21^. Differences in high-depth fragments were defined as SNPs or indels. In order to construct a high-quality genetic map, we filtered the SLAFs using five criteria: 1) removal of SLAFs from parents where the sequencing depth was less than 10X; 2) removal of SLAFs with more than three SNPs; 3) removal of SLAFs with the aa × bb segregation pattern; 4) removal of SLAFs with missing in more than 30% of offspring; 5) removal of segregation-distorted markers (p < 0.01). SLAFs that passed the five-step filtering process were used to construct a high-density genetic map. In this study, SLAFs followed the 1:1 Mendelian segregation pattern were used for genetic map construction. The generated SLAF markers were compared with the whole-genome sequences of perennial ryegrass (*Lolium perenne* L.), barley (*Hordeum vulgare* L.), rice (*Oryza sativa* L.) and *Brachypodium distachyon* by BLAST using an E-value cutoff of 1e-10 and an identity cutoff of 90% and requiring two paired matches.

SSR primers (Supplementary Table 7) were selected from several previous articles^14^,^15^. PCR reactions were performed according to Xie *et al*., with slight modifications^14^,^15^. Following are the conditions used: 5 min denaturation at 94 °C; 35 cycles of 1 min at 94 °C, 30 s at 52–58 °C, and 1 min at 72 °C; and then a final extension of 10 min at 72 °C and storage at 4 °C. The segregation data for SSR markers in the same population were detected, and 43 of them were used for constructing the genetic map.

### Map construction and QTL analysis

HighMap software, which consists of four modules–linkage grouping, marker ordering, error genotyping correction, and map evaluation^22^–was used for map construction. The single-linkage clustering algorithm was used to cluster the markers into linkage groups. The SLAF and SSR markers were ordered in the linkage groups, and genotyping errors were corrected using HighMap software. Based on the integrated map, significant loci associated with HD and FT were identified based on LOD scores larger than the 5% cutoff value determined through 1,000 permutation tests using the CIM method from “qtl” package of R. MapQTL6.0^55^ was used to conduct logarithm of odds and percentage of phenotypic variance explained analysis, and interval mapping (IM)^56^ was used to detect QTLs for target traits. HD and FT were investigated in 2014 and 2015 in Baoxing and Ya’an during the spring and summer seasons. HD and FT were recorded as the number of days from January 1st to the date when the first inflorescence fully emerged from the flag leaf and to the date of the first open floret on the plant, respectively^15^. The spearman correlation coefficient was analyzed by SAS.

## Conclusions

In conclusion, a high-density genetic linkage map was constructed with 2,467 SLAF markers and 43 SSR markers in orchardgrass. This map will serve as an invaluable tool for MAS in orchardgrass breeding programs. Comparative genomic analysis provided an overview of the relationship between orchardgrass and four related grass species. QTL analysis revealed stable and significant effects on flowering time and heading date phenotypes and provided valuable avenues for candidate gene exploration in the future. These data provide useful genomic resources for molecular dissection of complex traits, genome sequence assembly, and genetic improvement of the forage grass orchardgrass.

## Additional Information

**How to cite this article**: Zhao, X. *et al*. Construction of high-density genetic linkage map and identification of flowering-time QTLs in orchardgrass using SSRs and SLAF-seq. *Sci. Rep.* **6**, 29345; doi: 10.1038/srep29345 (2016).

## Supplementary Material

Supplementary Information

## Figures and Tables

**Table 1:** Marker information for high-density genetic map.

Linkage Group	Total Marker	Total Distance (cM)	Average Distance (cM)	Max Gap	SNP Number	SSR number
LG1	688	149.66	0.22	11.19	879	14
LG2	429	85.54	0.20	9.23	742	4
LG3	538	121.74	0.23	12.10	232	11
LG4	326	60.85	0.19	3.90	280	0
LG5	199	77.98	0.39	6.90	582	0
LG6	169	110.92	0.66	13.26	1,128	12
LG7	161	109.08	0.68	10.12	344	2
Total	2510	715.77	0.37	13.26	4,187	43

SNP, single nucleotide polymorphism; SSR, simple sequence repeat markers.

**Table 2:** Comparative genome analyses between orchardgrass and four other grass species.

	Total SLAF tags	Percentage	Mapped SLAF markers	Percentage
Total	447177		2467	
Rice	5432	1.21%	15	0.61%
*B. distachyon*	14478	3.24%	63	2.55%
Barley	19207	4.30%	73	2.96%
Perennial ryegrass	22448	5.02%	124	5.02%

**Table 3:** Descriptive statistics of phenotypic variation for flowering time and heading time in orchardgrass.

Trait	Environment	Min	Max	Mean ± SE	Skewness	Kurtosis	CV%
Flowering time	E1(2015-Baoxing)	102	145	118.93 ± 0.47	0.09	2.90	5.82%
	E2(2015-Ya’an)	72	140	99.66 ± 0.80	0.13	3.09	11.75%
	E3(2014-Hongya)	94	136	110.88 ± 0.67	0.09	2.90	8.81%
	E4(2014-Ya’an)	92	142	107.32 ± 0.53	0.06	2.67	7.22%
Heading time	E1(2015-Baoxing)	69	120	91.80 ± 0.45	−0.17	2.98	7.15%
	E2(2015-Ya’an)	53	115	82.38 ± 0.55	−0.13	3.04	9.79%
	E3(2014-Hongya)	61	120	90.46 ± 0.77	0.20	3.40	12.42%
	E4(2014-Ya’an)	55	117	83.89 ± 0.68	−0.11	3.37	11.83%

SE, standard error; CV, coefficient of variation.

**Table 4:** Spearman Correlation Coefficients in orchardgrass.

	BX15_HD	YA15_HD	HY14_HD	YA14_HD	BX15_FT	YA15_FT	HY14_FT
YA15_HD	0.50634**						
HY14_HD	0.55658**	0.53794**					
YA14_HD	0.53154**	0.55168**	0.59126**				
BX15_FT	0.87602**	0.52416**	0.53192**	0.55037**			
YA15_FT	0.46987**	0.76051**	0.48944**	0.61003**	0.54848**		
HY14_FT	0.49227**	0.52819**	0.91037**	0.58697**	0.55244**	0.54829**	
YA14_FT	0.48954**	0.51928**	0.6097**	0.82626**	0.59261**	0.64272**	0.66659**

BX15_HD, heading date at 2015 Baoxing, YA15_HD, heading date at 2015 Yaan, HY14_HD, heading date at 2014 Hongya, YA14_HD, heading date at 2014 Yaan; BX15_FT, flowering time at 2015 Baoxing, YA15_FT, flowering time at 2015 Yaan, HY14_FT, flowering time at 2014 Hongya, YA14_FT flowering time at 2014 Yaan.

**Table 5:** Flowering time and heading time QTLs identified in orchardgrass.

Trait	Environment	LG	Peak marker	LOD	Explanation (%)	Position
Flowering time	E1(2015-Baoxing)	5	Marker38787	4.11	27.00	4.72
		3	Marker139469	8.69	18.30	12.01
	E2(2015-Ya’an)	3	Marker167780	3.85	8.20	14.32
	E3(2014-Hongya)	3	Marker120144	6.55	13.50	2.99
	E4(2014-Ya’an)	3	Marker167780	6.45	13.40	14.32
Heading time	E1(2015-Baoxing)	3	Marker126472	12.21	23.60	18.31
		1	Marker45423	6.34	14.30	73.08
	E2(2015-Ya’an)	3	Marker167780	5.66	11.70	14.32
	E3(2014-Hongya)	3	Marker139469	5.47	13.20	12.01
		5	Marker38114	4.68	10.10	57.87
	E4(2014-Ya’an)	3	Marker167780	9.12	18.40	14.32

QTL, quantitative trait loci; LG, linkage group; LOD, likelihood of odds.
