# Supplementary material for: Construction of high-density genetic linkage map and identification of flowering-time QTLs in orchardgrass using SSRs and SLAF-seq
Source: Sci Rep. 2016 Jul 8;6:29345. doi: 10.1038/srep29345 (PMC4937404; doi:10.1038/srep29345)
Supplement: Supplementary Information [file srep29345-s1.pdf]

Construction of high-density genetic linkage map and identification of flowering-time QTLs in orchardgrass using SSRs and SLAF-seq

Xinxin Zhao[1#], Linkai Huang[1#], Xinquan Zhang[1*], Jianping Wang[2], Defei Yan[1], Ji Li[1], Lu Tang[1], Xiaolong Li[3] and Tongwei Shi[3].

1Department of Grassland Science, Sichuan Agricultural University, Chengdu, 611130, China,

2Agronomy Department, University of Florida, FL, 32610, USA,

3Biomarker Technologies Corporation, Beijing, 101300, China

*Corresponding author email: zhangxq@sicau.edu.cn.

#These authors contributed equally to this work.

Xinquan Zhang is the corresponding author and his information is as follows,

Department of Grassland Science, Animal Science and Technology College,

Sichuan Agricultural University

Huimin Road 211, Wenjiang, Chengdu 611130, Sichuan, China,

Tel: +86-28-86291780 (O) Fax: +86-28-86291010

E-mail: zhangxq@sicau.edu.cn

Running title: Orchardgrass genetic map and flowering-time QTLs

Keywords: Genetic linkage map, orchardgrass, QTL, flowering time, SLAF

Supplementary Table 1 Statistical analysis of sequencing data

Sample ID	Total Reads	Q30 Percentage（%）	GC Percentage（%）
P	11,667,001	89.40	44.99
M	11,702,366	89.46	45.04
offspring	1,251,603	89.34	45.22
control	93,742	89.29	46.46

Note: GC, guanine-cytosine

Supplementary Table 2 Orchardgrass SLAF tag information

Sample ID	SLAF Number	Total Depth	Average Depth
P	301,898	4,383,089	14.52
M	303,836	4,331,389	14.26
offspring	172,334	464,753	2.70

Supplementary Table 3 Orchardgrass SLAF types

Type	Polymorphic SLAF	Non-Polymorphic SLAF	Repetitive SLAF	Total SLAF
Number	89,038	349,362	8,777	447,177
Percentage	19.91%	78.13%	1.96%	100.00%

Supplementary Table 4 Orchardgrass SLAFs used for constructing genetic map

Type	SLAF Number	Percentage
efxeg	10	0.34%
hkxhk	115	3.94%
lmxll	1,225	41.92%
nnxnp	1,572	53.80%
Total	2,922	100.00%

Supplementary Table 5 Female genetic map information

Linkage Group ID	Total Marker	Total Distance(cM)	Average Distance(cM)	Max Gap
LG1	164	101.60	0.62	30.93
LG2	212	171.97	0.81	45.93
LG3	115	167.56	1.46	40.37
LG4	159	104.36	0.66	10.14
LG5	326	62.65	0.19	3.90
LG6	402	126.47	0.31	16.05
LG7	199	104.38	0.52	9.82
Total	1,577	838.99	0.65	45.93

Supplementary Table 6 Male genetic map information

Linkage Group ID	Total Marker	Total Distance(cM)	Average Distance(cM)	Max Gap
LG1	371	243.26	0.66	16.59
LG2	228	160.04	0.70	41.43
LG3	43	47.31	1.10	21.34
LG4	1	0.00	0.00	0.00
LG5	0	0.00	0.00	0.00
LG6	335	205.55	0.61	24.21
LG7	20	51.73	2.59	5.72
Total	998	707.89	0.81	41.43

Supplementary Table 7 Mapped SSR markers information

Marker names	Group	Forward Primer	Reverse Primer
MarkerFOG537N2	1	TAAATCTTGCACTTATCTGTGC	AACTGTACTCTCTCACACCCTC
MarkerFOG677N2	1	GGCACACGCCATTACTTC	TTCGTCGATGCAGTTGTC
MarkerA01L14N5	1	GCACAATGACACCAAATATG	ATCAGCATTGTGACCACC
MarkerOGA148N2	1	AACTTCGAACACATACATAGCA	TAGTCTGTCAGTGTCGTGGA
MarkerD03P02N6	1	TGTAGAACAATCCATCTTTGG	TCATGAGATCTAGCGGTTTT
MarkerB02I20N5	1	CATGAGAGCAAGAACATTAAGA	AACACATGGAAGGATAGCAC
MarkerFOG21N3	1	CTAAATCTTAGCTGGGCAAA	ACCAAATCAGCCAAAACTC
MarkerB06H11N3	1	GCCAGACAGAGTGGTGAG	ATTGATGATGCTACACTCCC
MarkerA01L14N1	1	GCACAATGACACCAAATATG	ATCAGCATTGTGACCACC
MarkerFOG210N1	1	CTAAATCTTAGCTGGGCAAA	ACCAAATCAGCCAAAACTC
MarkerD03P02N2	1	TGTAGAACAATCCATCTTTGG	TCATGAGATCTAGCGGTTTT
MarkerC01K17N2	1	TTAGATGCATTAGACTCCACG	ACTTCTCCAAATGGCAATC
MarkerB05P02N4	1	AGCAGATTGAAGTGGAGAGA	TACCCTTCACACTCCACG
MarkerB06H11N1	1	GCCAGACAGAGTGGTGAG	ATTGATGATGCTACACTCCC
MarkerB06C05N3	2	ACAGATGCATGCTTATGTGA	AATTGCCATGAATGTTGG

MarkerFOG365N1	2	GGTGCAGGATTTACATCAGT	AAATTAGGCAAAATAACACACC
MarkerFOG365N3	2	GGTGCAGGATTTACATCAGT	AAATTAGGCAAAATAACACACC
MarkerB02F03N7	2	GCAACAAGTAGTTGAGCACA	TCGACTCTGATGCCAATT
MarkerB04P11N2	3	ACGAGAGCAATCTCCTATCA	TATGGGAAGGGCTCTCTC
MarkerB05C01N1	3	GTGATACGACTGCAAATCG	ATATCGTATCCGAAATCATCTC
MarkerA02A10N1	3	AGGTTACCGATAGTAAGTGGG	AGGGGATGGTTGGTTAGTAT
MarkerB02B19N11	3	ATCGGCCCGCGAGAAC	CACCACACTTCCTCCACTCA
MarkerFOG515N1	3	GATGAAGGAACTGCTGGAT	ACACCAGACCCTAAACAGC
MarkerB02B19N7	3	ATCGGCCCGCGAGAAC	CACCACACTTCCTCCACTCA
MarkerB02B19N6	3	ATCGGCCCGCGAGAAC	CACCACACTTCCTCCACTCA
MarkerB04C12N3	3	GTTTCATCTCTAAACTGAAGGC	AACAACTGGGATGTCTTCAC
MarkerB05C01N2	3	GTGATACGACTGCAAATCG	ATATCGTATCCGAAATCATCTC
MarkerB04C12N4	3	GTTTCATCTCTAAACTGAAGGC	AACAACTGGGATGTCTTCAC
MarkerFOG531N1	3	CGCATATGTGAAGAACAAGA	AAGTCAGACGACCCATCTC
MarkerFOG515N3	6	GATGAAGGAACTGCTGGAT	ACACCAGACCCTAAACAGC
MarkerB02P24N5	6	GGGGAGGTACCCACTTCT	TTACCCAATCTAAGATCTTTGC
MarkerC01C17N2	6	CAAATACGTTACGTCCCG	CCGAAGATACGATCGAACTA
MarkerA01K14N5	6	AAGGATGGCCTGATCTTC	GCAGAGGTCTTTCTCTTGG
MarkerA03K22N1	6	AGACTCTAGGGTGGCACAC	GTAGCACGCTAACGAGAGAT
MarkerA03K22N4	6	AGACTCTAGGGTGGCACAC	GTAGCACGCTAACGAGAGAT
MarkerC01C17N1	6	CAAATACGTTACGTCCCG	CCGAAGATACGATCGAACTA
MarkerA01K14N4	6	AAGGATGGCCTGATCTTC	GCAGAGGTCTTTCTCTTGG
MarkerC01J21N5	6	GAGGAGGCTGGAAGAGAG	ATCTTGGATCTAATGGAATTTG
MarkerA01L12N3	6	GGCTCAATCCTTAGACACTG	ACGAGAAATCGTCGTATTGT
MarkerFOG402N2	6	TCCTTATGAAATGAATGAATGA	AAGAACTGGACATATACTTGGG
MarkerC01C17N3	6	CAAATACGTTACGTCCCG	CCGAAGATACGATCGAACTA
MarkerFOG634N2	7	GTGCGTCTTTTAAATGGTATG	GAGCCTCCCTAACCCTAGTA
MarkerB03F08N2	7	TGAGGTGCTTTATATATGGTGA	AATTAGTCATCGAGGTTTATGC

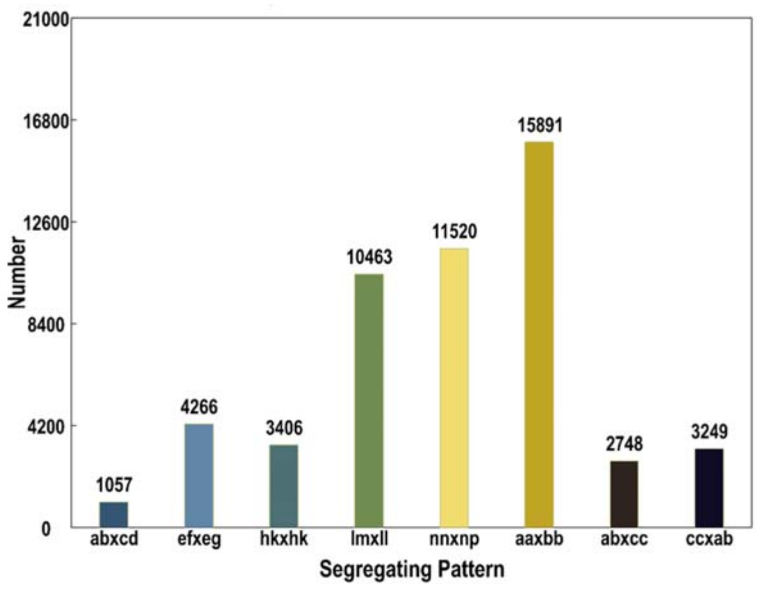

Supplemental Figure 1 Genotype distribution of SLAF markers

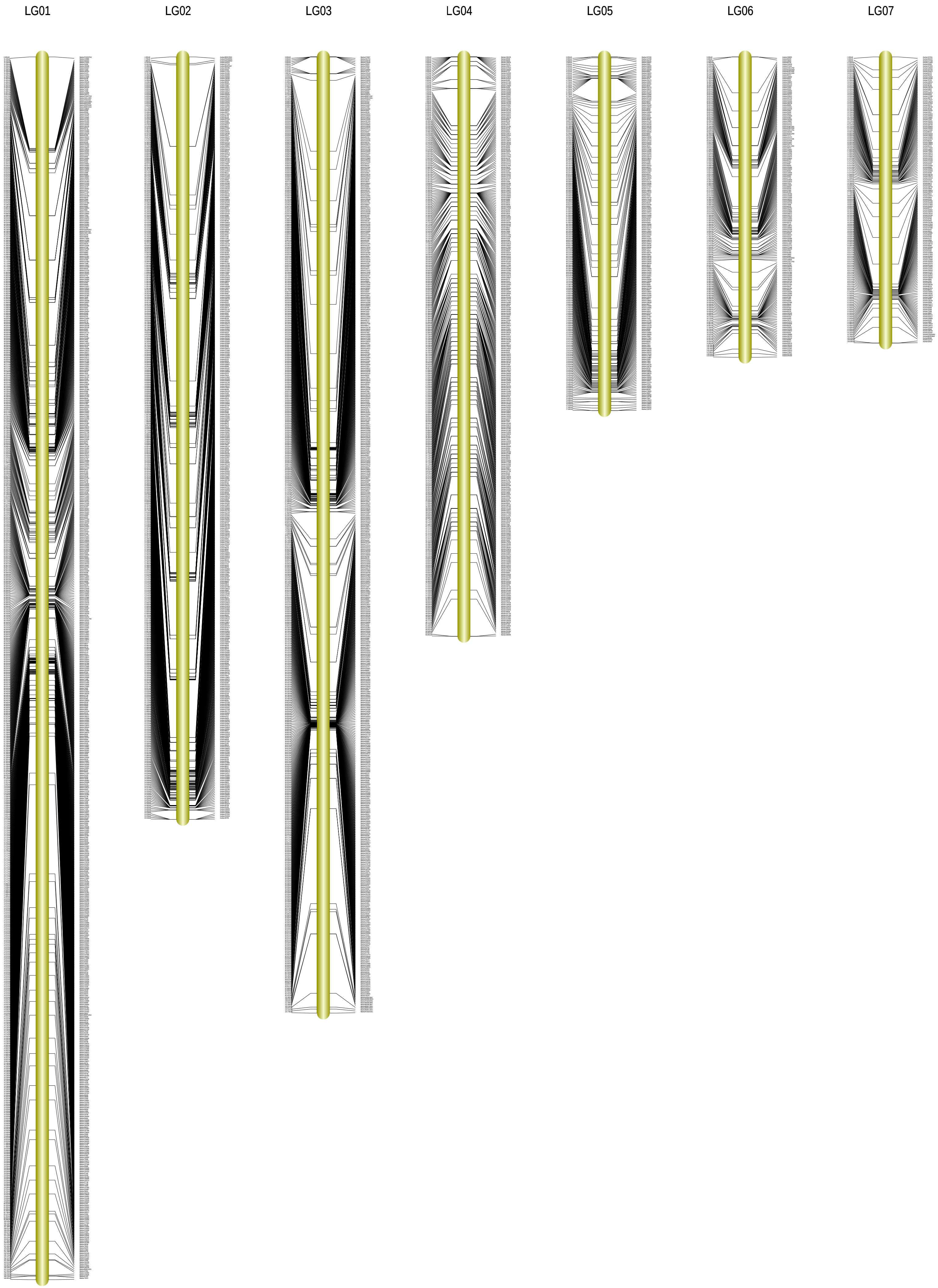
LG01
LG02
LG03
LG04
LG05
LG06
LG07

Supplemental Figure 2 High-density genetic map marker information

a

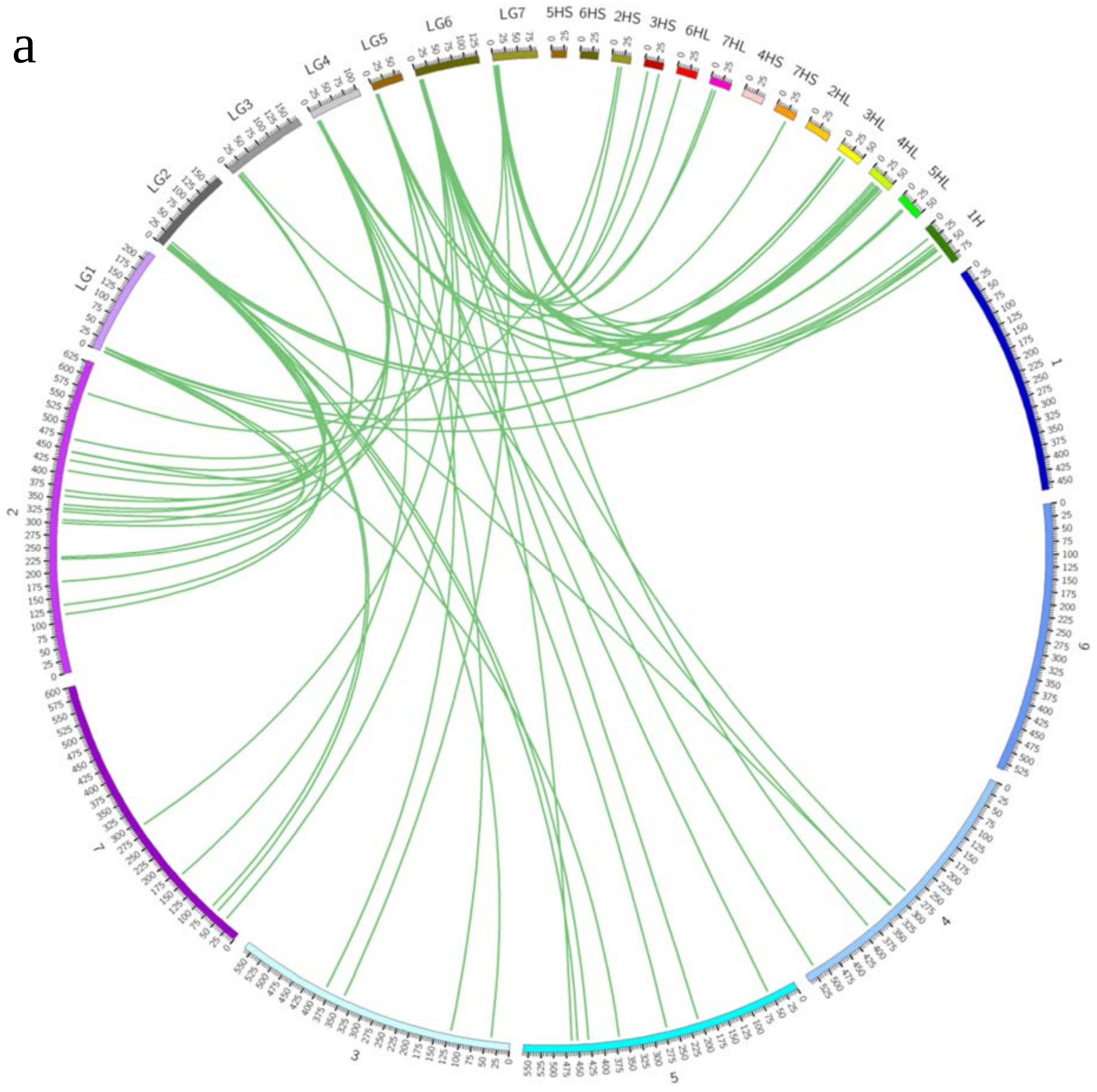

b

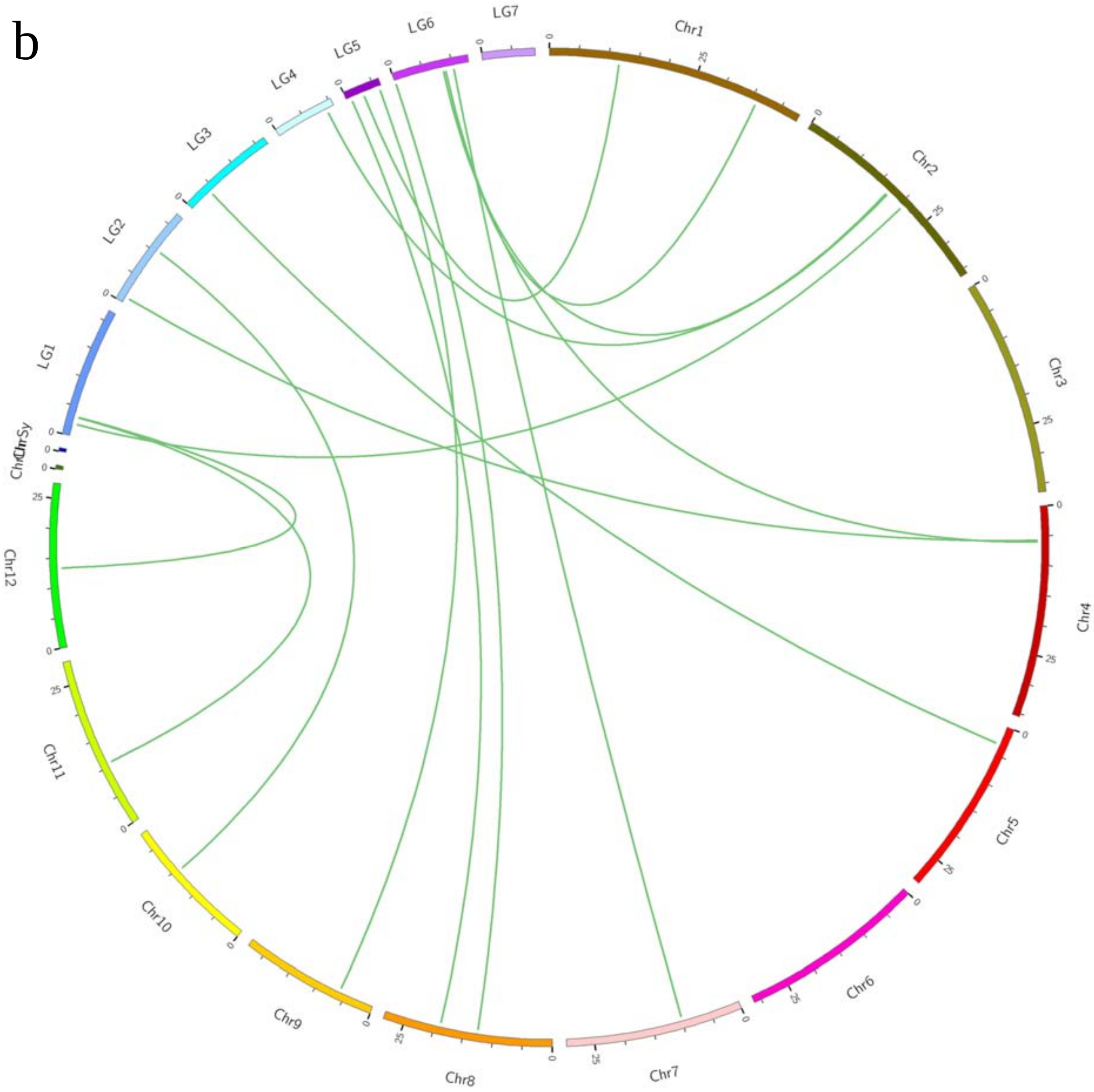
LG1
LG2
LG3
LG4
LG5
LG6
LG7
Chr1
Chr2
Chr3
Chr4
Chr5
Chr6
Chr7
Chr8
Chr9
Chr10
Chr11
Chr12

c

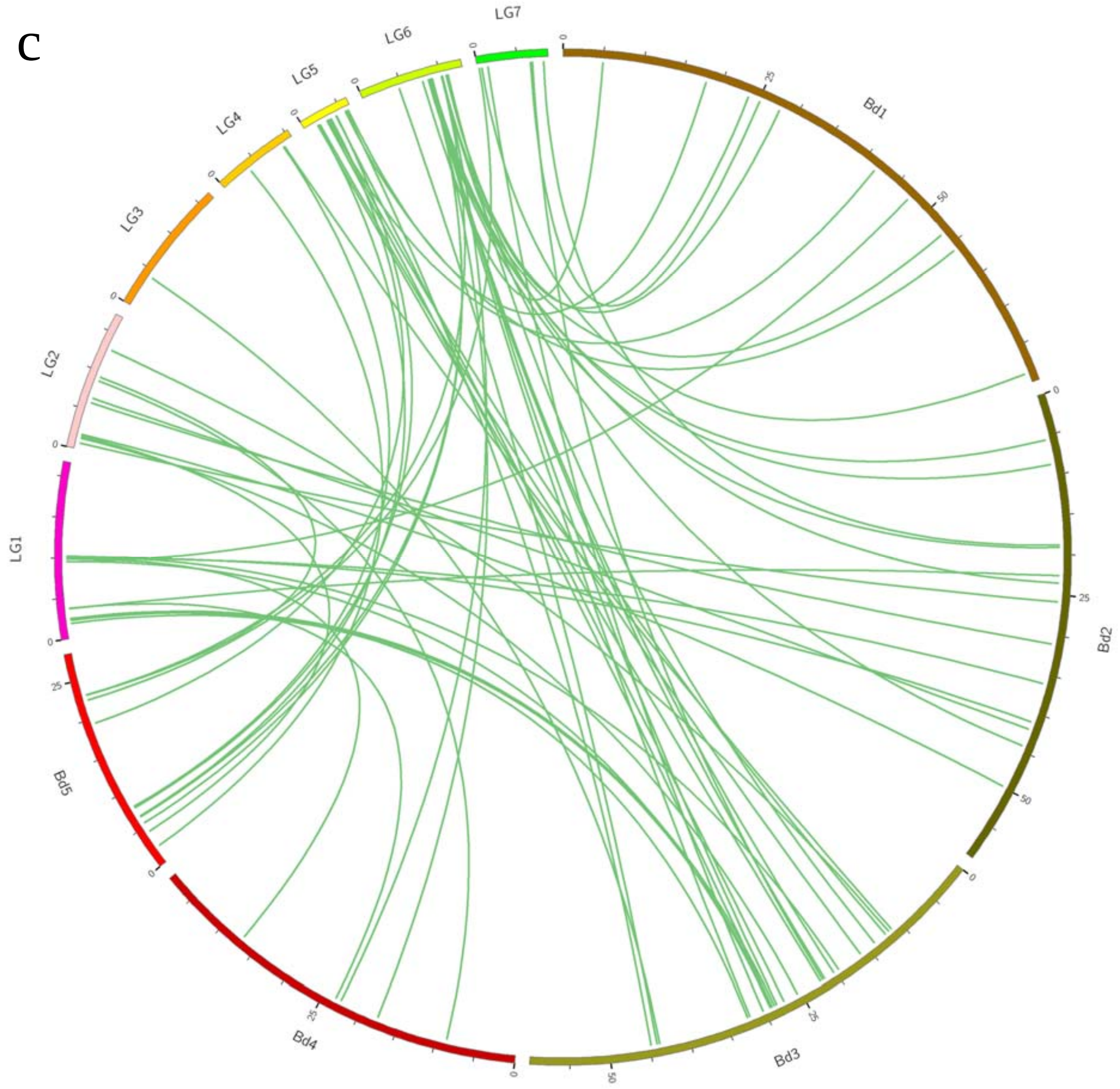

Supplemental Figure3 Comparison between orchardgrass and three other grass genomes.

Circos plot showing linear relationship between orchardgrass and barley (a), rice (b) and *Brachypodium distachyon*(c)
